# Efficacy and Safety of Sinomenine Preparation for Ankylosing Spondylitis: A Systematic Review and Meta-Analysis of Clinical Randomized Controlled Trials

**DOI:** 10.1155/2020/4593412

**Published:** 2020-05-14

**Authors:** Shan-Shan Lin, Chun-Xiang Liu, Jun-Hua Zhang, Hui Wang, Jing-Bo Zhai, Jing-Yuan Mao, Xian-Liang Wang

**Affiliations:** ^1^First Teaching Hospital of Tianjin University of Traditional Chinese Medicine, Tianjin 300381, China; ^2^Evidence-Based Medicine Center, Tianjin University of Traditional Chinese Medicine, Tianjin 301617, China

## Abstract

**Objectives:**

To systematically evaluate the efficacy and safety of sinomenine preparation (SP) for treating ankylosing spondylitis (AS).

**Methods:**

Clinical randomized controlled trials (RCTs) of SP for treating AS were systematically identified in six electronic databases including PubMed, Embase, Cochrane Library, China National Knowledge Infrastructure (CNKI), Chinese Scientific Journal Database (VIP), and Wanfang Databases from the inception up to 31 October 2019. Cochrane's risk of bias tool was used to assess the methodological quality and Review Manager 5.3 software was used to analyze data.

**Results:**

A total of 12 RCTs involving 835 patients were finally included. According to interventions, RCTs were divided into two types. The intervention in 10 RCTs was SP combined with conventional pharmacotherapy (CPT) versus CPT and that in 2 RCTs was SP alone versus CPT. The results of the meta-analysis showed that, compared with CPT alone, SP combined with oral CPT has better improvement in BASDAI (WMD = −1.84, 95% CI [−3.31, −0.37], *P*=0.01), morning stiffness time (WMD = −13.46, 95% CI [−16.12, −10.79], *P* < 0.00001), the Schober test (WMD = 1.26, 95% CI [0.72, 1.80], *P* < 0.00001), the occipital wall test (WMD = −0.55, 95% CI [−0.96, −0.14], *P*=0.009), the finger-to-ground distance (WMD = −3.28, 95% CI [−5.64, −0.93], *P*=0.006), 15 m walking time (WMD = −8.81, 95% CI [−13.42, −4.20], *P*=0.0002), the C-reactive protein (CRP) (WMD = −1.84, 95% CI [−3.24, −0.45], *P*=0.01), and the total effective rate (RR = 1.10, 95% CI [1.01, 1.20], *P*=0.03). Besides, it also showed that oral SP alone may be more effective in improving morning stiffness time (WMD = −31.89, 95% CI [−34.91, −28.87], *P* < 0.00001) compared with CPT alone. However, this study cannot provide evidence that loading the injectable SP based on CPT can significantly increase the efficacy due to the insufficient number of studies included. In terms of adverse events, there was no statistically significant difference between the experimental group and the control group.

**Conclusions:**

This study shows that oral SP may be effective and safe in the treatment of AS. Due to the low methodological quality of the included RCTs and the limitations of the meta-analysis, it is still necessary to carry out more multicenter, large-sample, and high-quality RCTs to further verify the conclusions. The review protocol was registered on PROSPERO (CRD42018099170), and the review was constructed following the PRISMA guidelines (Annex 1).

## 1. Introduction

Axonal spinal arthritis (axSpA) is a clinically common chronic progressive inflammatory disease caused by cellular stresses [1, 2]. Ankylosing spondylitis (AS) is a representative disease of axSpA, accompanied by structural damage to the sacroiliac joints (such as narrowing of joint space, erosion, and subchondral bone sclerosis) observed by X-ray examinations [3, 4]. In severe cases, spinal rigidity, deformity, and dysfunction have even occurred and seriously affected the quality of life of patients [5]. AS is more common in young men, and it is one of the major causes of the loss of labor force in China's young and middle-aged people [6]. Recent investigations suggest that disease pathogenesis is ascribed to a complex interplay of genetic, environmental, endocrine disorders, and autoimmune function [7, 8].

Several studies demonstrated that the persistence of inflammation is an important predisposing factor of subsequent structural articular damage [9, 10]. Until now, there is no available method for early diagnosis of AS. And the effective therapy for AS stays largely undefined. Experts in related fields believe that the main therapeutic goal of AS is to prevent the progressive structural damage by controlling symptoms and inflammation to maintain the body function of the patient, thereby maximizing social participation and improving long-term quality of life [11]. According to the guidelines for the treatment of axial spondyloarthritis developed by the American College of Rheumatology in 2016 [11], the optimal management of AS requires a combination of pharmacological and nonpharmacological treatment models. At present, the first-line drugs for treating AS are nonsteroidal anti-inflammatory drugs (NSAIDs) [12], which are often supplemented with antirheumatic drugs, glucocorticoids, biological agents, and traditional Chinese medicine (TCM) [13, 14]. Nonpharmacological treatments include physiotherapy, physical exercise, and health education for patients and their families [13, 14]. The serious complications of advanced AS include hip fusion, spinal deformity, and a spinal fracture. If necessary, surgeries such as total hip arthroplasty and spine surgery should be performed. AS is a chronic disease requiring long-term medication. The long-term medication will cause serious adverse reactions and economic burden [15, 16].

Besides, many meta-analyses have shown an increased risk of cardiovascular events associated with NSAIDs [17–19]. Therefore, it is still urgently required to look for effective, safe, and cost-effective treatment methods that can treat AS through other mechanisms [20].

In TCM, the plant *Sinomenium acutum* (in China known as Fang-ji or Qing-feng-teng), a Chinese herbal medicine, is widely used to treat rheumatic arthritis and has the advantages of mild toxicity and no addiction [21]. Sinomenine is an alkaloid originally isolated from the root of *Sinomenium acutum*. Its medicinal form is generally hydrochloride [22]. Studies have shown that sinomenine has anti-inflammatory [23, 24], immunosuppression [25], cartilage protection [26, 27], vascular endothelium protection [28], analgesic [29], and other effects. At present, sinomenine preparation (SP) approved by the State Food and Drug Administration of China includes Zhengqing Fengtongning Conventional Tablet, Zhengqing Fengtongning Sustained Release Tablet, Zhengqing Fengtongning Enteric-Coated Tablet, Zhengqing Fengtongning Capsule, and Zhengqing Fengtongning Injection. Many clinical trials have shown that SP has a good therapeutic effect on AS [30, 31]. Therefore, as a modern preparation, SP is expected to become an emerging and special medicine for the treatment of AS and has broad prospects [32, 33]. Because the sample size of a single study is too small, the level of evidence for the efficacy and safety of the SP in the treatment of AS is very low. Therefore, it is necessary to conduct a systematic review and meta-analysis to evaluate the efficacy and safety of SP in the treatment of AS, to objectively provide a reference for the rational use of drugs and individual treatment in the clinic.

## 2. Methods

### 2.1. Selection Criteria

#### 2.1.1. Study Design

Clinical randomized controlled trials (RCTs) of SP for the treatment of AS were included.

#### 2.1.2. Participants

Studies indicated that the diagnostic criteria were nationally recognized New York classification criteria for AS [3]. And participants did not have serious complications. There were no restrictions on participants' gender, age, race, duration of disease, source of the case, and follow-up time.

#### 2.1.3. Interventions

The experimental group used sinomenine preparation (SP) alone or SP combined with conventional pharmacotherapy (CPT). The type of preparation and the route of administration are not limited. The SP included Zhengqing Fengtongning Conventional Tablet, Zhengqing Fengtongning Sustained Release Tablet, Zhengqing Fengtongning Enteric-Coated Tablet, Zhengqing Fengtongning Capsule, and Zhengqing Fengtongning Injection. The control group used CPT alone. The CPT included NSAIDs, antirheumatic drugs, immunosuppressants, and biological agents. Both the experimental group and the control group were not combined with other traditional Chinese medicine prescriptions, Chinese herbal compound decoctions, external dressings, or fumigation lotions containing sinomenine extract.

#### 2.1.4. Outcomes

Studies using at least one of the following outcomes were included.

*(1) Primary Outcomes*. Recognized standardized scales: bath ankylosing spondylitis disease activity index (BASDAI) [34] and bath ankylosing spondylitis functional index (BASFI) [35].

*(2) Secondary Outcomes*.

  ① Clinical symptom measures: morning stiffness time, the Schober test, chest expansion, occipital wall test, finger-to-ground distance, and 15 m walking time
  ② Laboratory measures: erythrocyte sedimentation rate (ESR) and C-reactive protein (CRP) play an important role in judging the severity of AS [10, 36, 37]
  ③ Total effective rate depending on the degree of improvement of clinical symptoms and laboratory measures
  ④ Adverse reactions

#### 2.1.5. Exclusion Criteria

  ① Interventions include other TCM treatments, such as traditional Chinese medicine decoction, other Chinese patent medicine, acupuncture, and rehabilitation therapy
  ② After seeking help online or contacting the corresponding author via e-mail, studies whose full text cannot be obtained need to be excluded
  ③ Studies that do not provide data for synthesis will be excluded
  ④ For studies that repeatedly published by different centers, we only included the one with the most complete results and the highest quality
  ⑤ Unfinished protocol, case report, systematic review, and meta-analysis were excluded

### 2.2. Search Strategy

The clinical RCTs about SP in the treatment of AS were searched in the relevant database, including PubMed, Embase, Cochrane Library (No. 10 of 2019), China National Knowledge Infrastructure (CNKI), Wan Fang database, and VIP database. The retrieval time was from inception to October 2019. The search terms mainly included Zhengqing Fengtongning, Sinomenine, ankylosing spondylitis, randomized controlled trial, and their synonyms. The search strategies combining medical subject headings (MeSH) and free-text terms were used. Different search strategies were adopted according to the characteristics of each database. The synonyms in every group are connected by “or,” and the search terms between the groups are connected by “and.” At the same time, the reference lists of existing systematic reviews and meta-analyses were further searched to avoid omissions. The language of the literature was not limited. The development and implementation of the search strategy were completed by a clinician Lin and a methodologist Liu and revised by the library staff. The detailed search strategy of PubMed as an example was shown in Table 1.

### 2.3. Data Extraction and Quality Evaluation

The data extraction contents mainly included sample data, diagnostic criteria, interventions, efficacy criteria, outcomes, and adverse reactions. Finally, according to the Cochrane's risk of bias tool including random sequence generation, allocation concealment, blinding of participants and personnel, blinding of outcome assessment, incomplete outcome data, selective reporting, and other sources of bias, the methodological qualities of the included studies were assessed with “low” (representing the low risk of bias), “unclear” (for medium or unknown risk of bias), or “high” (for high risk of bias) [38]. Data extraction and quality evaluation were performed by two reviewers (Lin and Liu) independently according to the selection criteria, and disagreements were resolved through discussion with the third reviewer (Zhang).

### 2.4. Statistical Analysis

Data analysis was performed using Review Manager 5.3 software. The risk ratio (RR) or odds ratio (OR) with a 95% confidence interval (CI) was calculated for dichotomous outcomes. The weighted mean difference (WMD) or standardized mean difference (SMD) with 95% CI was calculated for continuous outcomes. *P* < 0.05 was considered to be statistically significant. The statistical heterogeneity was estimated according to *I*^2^ statistics. If the results had no statistical heterogeneity (*P*≧0.1, *I*^2^ ≦ 50%), a fixed-effect model would be used to combine analysis. When there was statistical heterogeneity (*P* < 0.1, *I*^2^ > 50%), the subgroup analysis would be needed if there was obvious clinical or methodological heterogeneity. If no obvious clinical or methodological heterogeneity was found, a random-effect model would be used. For the results of the original study that could not be used for meta-analysis, we would perform a qualitative descriptive analysis. If the number of studies included was sufficient (≧10 articles), a funnel plot would be drawn to analyze for potential publication bias.

## 3. Results

### 3.1. Literature Search Result

**Figure 1:**
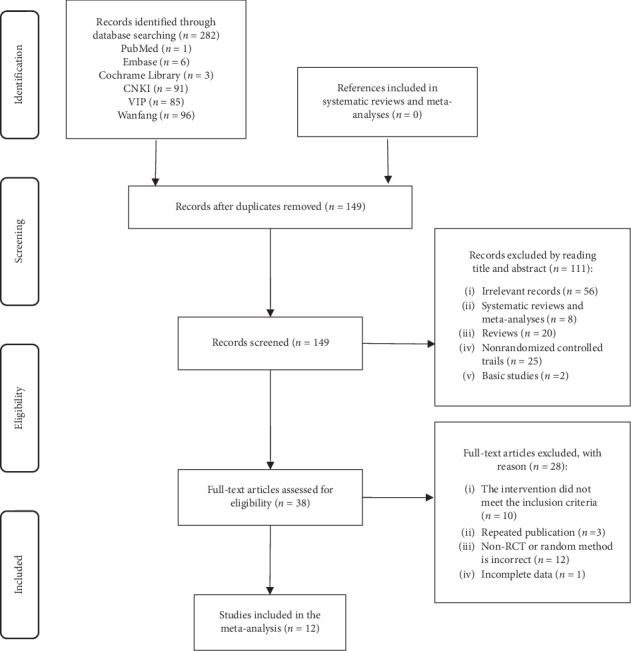
Flowchart of the literature selection process.

We identified a total of 282 studies of the potentially relevant literature in the initial retrieval. Firstly, we read the title and abstract to exclude the literature whose content was irrelevant. Secondly, we read the full text to determine whether it would be eventually included. Finally, twelve studies [39–50] published from 2001 to 2014 were included. The literature screening process and the result were shown in Figure 1.

### 3.2. Basic Conditions of the Included Studies

Twelve RCTs involving 835 patients were included. According to the interventions, the included studies were divided into two types of treatment comparisons. The first type was SP combined with CPT (SP + CPT) versus CPT in 10 RCTs. The second type was SP alone versus CPT in 2 RCTs. CPT included sulfasalazine, meloxicam, methotrexate, thalidomide, inflammatory pain tablet, vitamins, and ranitidine. The duration of the treatment was one month to 18 months. See Table 2 for details.

### 3.3. Quality Assessment of Included Studies

**Figure 2:**
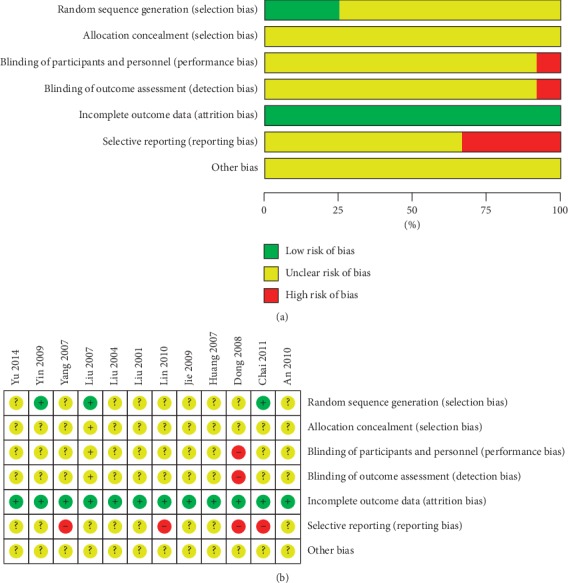
(a) Risk of bias graph. (b) Risk of bias summary.

All studies used randomization, but only three studies [44, 49, 50] detailed that the specific method of random sequence generation was to use a random number table. We considered them to be low risk. None of the studies mentioned allocation concealment. Almost all the studies did not mention the blinding of participants and outcome assessment. However, one study [41] was a prospective-open-controlled trial, which indicated that blinding was not used. We considered that both the risk of performance bias and the risk of detection bias were high. None of the studies had attrition bias due to incomplete outcome data. In terms of selective reporting, three studies [42, 45, 50] did not report all of the preset outcomes in the results section, such as radiographs of the sacroiliac joint, lumbar spine, and chest. In one study [41], although the results were tested multiple times, only one of them was reported. Therefore, we considered them [41, 42, 45, 50] to be a high risk of reporting bias. All studies did not have sufficient evidence to support the existence of other biases. Overall, the quality of 10 studies is low or remains unclear due to the high proportion of the unclear risk of biases in most studies. The results of the quality assessment are summarized in Figure 2.

### 3.4. Data Analyses of SP + CPT versus CPT

The first type of intervention comparisons was SP + CPT versus CPT, including ten studies [39–48]. The results of data analyses are as follows.

#### 3.4.1. Clinical Symptom Measures

**Figure 3:**
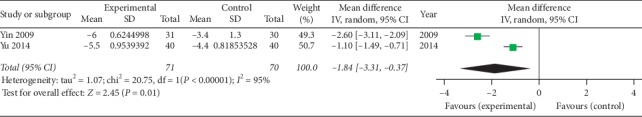
Forest plot of BASDAI of oral SP + CPT versus CPT.

*(1) BASDAI*. Two of these studies [44, 46] used the internationally recognized BASDAI scoring standard. Because of the significant heterogeneity (*P* < 0.00001, *I*^2^ = 95%), a random-effect model was used.  Figure 3 shows that oral SP + CPT may be more beneficial to reduce the BASDAI than CPT in treating AS (WMD = −1.84, 95% CI [−3.31, −0.37], *P*=0.01). Only one study [47] using injectable SP + CPT reported BASDAI, so no meta-analysis was performed. The experimental group used injectable SP (intra-articular injection, 35 ml, once a week) based on the treatment of the control group. Jie et al. [47] thought that there was no statistically significant difference between injectable SP + CPT and CPT alone in the improvement of BASDAI (*P* > 0.05).

*(2) BASFI*. Two studies [44, 47] used the internationally recognized BASFI scoring standard. Yin et al. [44] thought that loading sinomenine hydrochloride sustained-release tablets based on sulfasalazine and meloxicam may reduce the BASFI more effectively (*P*=0.002). Jie et al. [47] thought that loading Zhengqing Fengtongning Injection based on CPT may reduce the BASFI more effectively (*P* < 0.05).

**Figure 4:**
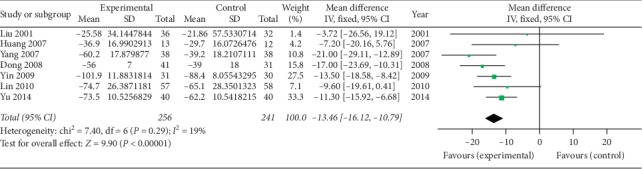
Forest plot of morning stiffness time of oral SP + CPT versus CPT.

*(3) Morning Stiffness Time (min)*. Seven studies [39, 41–46] using oral SP + CPT reported morning stiffness time.  Figure 4 shows a statistically significant difference, indicating that oral SP + CPT for treating AS may be more beneficial to shorten the morning stiffness time than CPT (WMD = −13.46, 95% CI [−16.12, −10.79], *P* < 0.00001).

**Figure 5:**
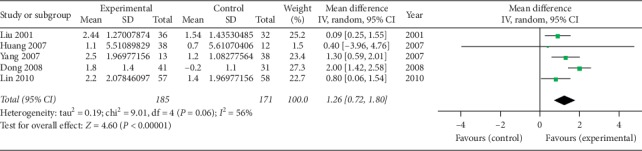
Forest plot of the oral Schober test of oral SP + CPT versus CPT.

*(4) The Schober Test (cm)*. Five studies [39, 41–43, 45] using oral SP + CPT reported the Schober test. Because of the significant heterogeneity (*P*=0.06, *I*^2^ = 56%), a random-effect model was used.  Figure 5 shows that oral SP + CPT may be more beneficial to improve the Schober test than CPT in treating AS (WMD = 1.26, 95% CI [0.72, 1.80], *P* < 0.00001). One study [47] using injectable SP + CPT reported the Schober test. Jie et al. [47] thought that there was no statistically significant difference between injectable SP + CPT and CPT alone in the improvement of the Schober test (*P* > 0.05).

**Figure 6:**
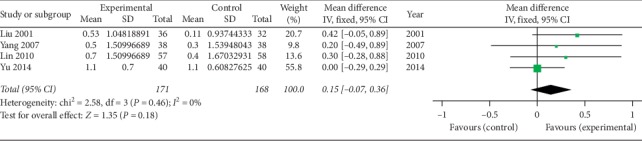
Forest plot of chest expansion of oral SP + CPT versus CPT.

*(5) Chest Expansion (cm)*. Four studies [39, 42, 45, 46] using oral SP + CPT reported the chest expansion.  Figure 6 shows that it has not been proven that there is a statistically significant difference in the improvement of the chest expansion between oral SP + CPT and CPT (*P*=0.18). One study [47] using injectable SP + CPT reported the chest expansion. Jie et al. [47] thought that there was no statistically significant difference between injectable SP + CPT and CPT alone in the improvement of the chest expansion (*P* > 0.05).

**Figure 7:**
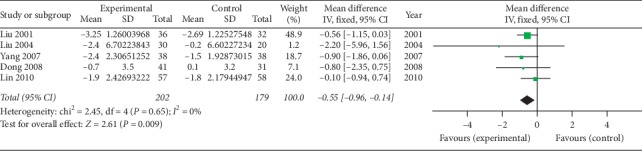
Forest plot of occipital wall test of oral SP + CPT versus CPT.

*(6) Occipital Wall Test (cm)*. Five studies [39–42, 45] using oral SP + CPT reported the occipital wall test.  Figure 7 shows that the difference is statistically significant, indicating that oral SP + CPT may be more beneficial to improve the symptoms of the occipital wall test than CPT in treating AS (WMD = −0.55, 95% CI [−0.96, −0.14], *P*=0.009).

**Figure 8:**
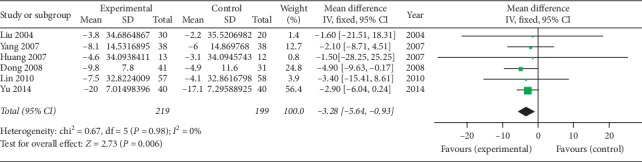
Forest plot of the finger-to-ground distance of oral SP + CPT versus CPT.

*(7) Finger-to-Ground Distance (cm)*. Six studies [40–43, 45, 46] using oral SP + CPT reported finger-to-ground distances.  Figure 8 shows that the difference is statistically significant, indicating that oral SP + CPT may be more beneficial to improve the finger-to-ground distance than CPT in treating AS (WMD = −3.28, 95% CI [−5.64, −0.93], *P*=0.006).

**Figure 9:**
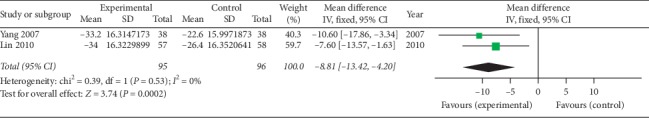
Forest plot of 15 m walking time of oral SP + CPT versus CPT.

*(8) 15* m *Walking Time (s)*. Two studies [42, 45] using oral SP + CPT reported 15 m walking time.  Figure 9 shows that the difference is statistically significant (WMD = −8.81, 95% CI [−13.42, −4.20], *P*=0.0002). Current evidence shows that oral SP + CPT for treating AS may be more beneficial to shorten the 15 m walking time than CPT.

#### 3.4.2. Laboratory Measures

**Figure 10:**
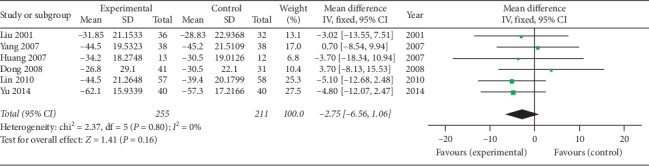
Forest plot of ESR of oral SP + CPT versus CPT.

*(1) ESR (mm/h)*. Six studies [39, 41–43, 45, 46] using oral SP + CPT reported ESR.  Figure 10 shows that it has not been proven that there is a statistically significant difference in the improvement of ESR between oral SP + CPT and CPT (*P*=0.16). One study [47] using injectable SP + CPT reported ESR. Jie et al. [47] thought that there was no statistically significant difference between injectable SP + CPT and CPT alone in the improvement of ESR (*P* > 0.05).

**Figure 11:**
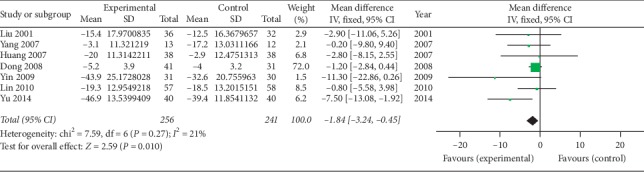
Forest plot of CRP of oral SP + CPT versus CPT.

*(2) CRP (mg/l)*. Seven studies [39, 41–46] using oral SP + CPT reported CRP.  Figure 11 shows that oral SP + CPT may be more beneficial to improve CRP than CPT alone (WMD = −1.84, 95% CI [−3.24, −0.45], *P*=0.01). One study [47] using injectable SP + CPT reported CRP. Jie et al. [47] thought that there was no statistically significant difference between injectable SP + CPT and CPT alone in the improvement of CRP (*P* > 0.05).

#### 3.4.3. Total Effective Rate

**Figure 12:**
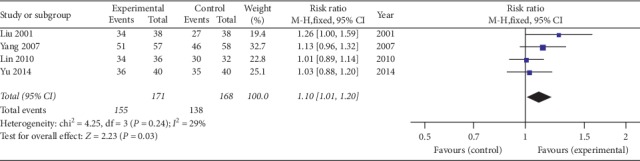
Forest plot of total effective rate of oral SP + CPT versus CPT.

Nine studies [39–47] all reported the total effective rate, four of which [39, 42, 45, 46] had consistent efficacy criteria. The effective criteria were at least 50% improvement in outcome measures such as ESR, CRP, morning stiffness time, number of joint swellings and pains, the Schober test, and chest expansion. Those who had achieved at least 3 criteria were effective as a whole; otherwise, they were ineffective.  Figure 12 shows that the difference is statistically significant, indicating that oral SP + CPT may be more beneficial to improve the total effective rate than CPT in treating AS (RR = 1.10, 95% CI [1.01, 1.20], *P*=0.03).

### 3.5. Data Analyses of SP Alone versus CPT

The second type of intervention comparison was SP alone versus CPT, including two studies [49, 50]. The results of data analyses are as follows.

#### 3.5.1. Clinical Symptom Measures

**Figure 13:**
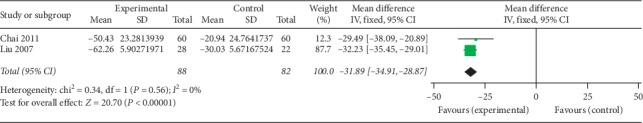
Forest plot of morning stiffness time of SP alone versus CPT.

*(1) Morning Stiffness Time (min)*. Both studies [49, 50] reported the morning stiffness time.  Figure 13 shows that the difference is statistically significant (WMD = −31.89, 95% CI [−34.91, −28.87], *P* < 0.00001). Current evidence shows that oral SP alone may be more beneficial in shortening the morning stiffness time of AS patients than sulfasalazine.

**Figure 14:**
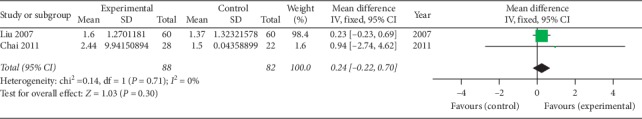
Forest plot of the Schober test of SP alone versus CPT.

*(2) The Schober Test (cm)*. Both studies [49, 50] reported the Schober test.  Figure 14 shows that the difference is not statistically significant (WMD = 0.24, 95% CI [−0.22, 0.70], *P*=0.30). It has not been proven that there is a statistically significant difference in improving the symptoms of the Schober test between oral SP alone and sulfasalazine.

**Figure 15:**
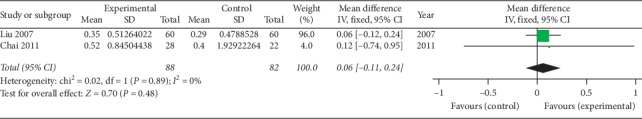
Forest plot of chest expansion of SP alone versus CPT.

*(3) Chest Expansion (cm)*. Both studies [49, 50] reported chest expansion.  Figure 15 shows that the difference is not statistically significant (WMD = 0.06, 95% CI [−0.11, 0.24], *P*=0.48). It has not been proven that there is a statistically significant difference in improving chest expansion between oral SP alone and sulfasalazine.

**Figure 16:**
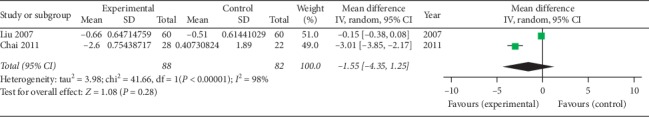
Forest plot of occipital wall test of SP alone versus CPT.

*(4) Occipital Wall Test (cm)*. Both studies [49, 50] reported the occipital wall test. Because of the significant heterogeneity between the two studies (*P* < 0.00001, *I*^2^ = 98%), a random-effect model was used.  Figure 16 shows that the difference is not statistically significant (WMD = −1.55, 95% CI [−4.35, 1.25], *P*=0.28). It has not been proven that there is a statistically significant difference in improving the occipital wall test between oral SP alone and sulfasalazine.

*(5) Finger-to-Ground Distance (cm)*. One study [49] reported finger-to-ground distance. The study [49] showed that there was no statistically significant difference between oral SP alone and sulfasalazine in improving the finger-to-ground distance (*P*=0.17).

#### 3.5.2. Laboratory Measures

**Figure 17:**
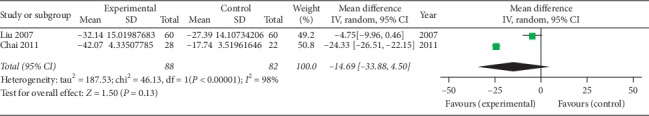
Forest plot of ESR of SP alone versus CPT.

*(1) ESR (mm/h)*. Both studies [49, 50] using SP alone reported ESR. Because of the significant heterogeneity between the two studies (*P* < 0.00001, *I*^2^ = 98%), a random-effect model was used.  Figure 17 shows that the difference is not statistically significant (WMD = −14.69, 95% CI [−33.88, 4.50], *P*=0.13). It has not been proven that there is a statistically significant difference in improving ESR between oral SP alone and sulfasalazine.

**Figure 18:**
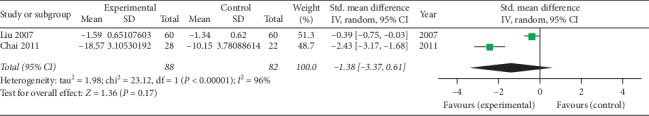
Forest plot of CRP of SP alone versus CPT.

*(2) CRP (mg/l)*. Both studies [49, 50] using SP alone reported CRP. Since there was a methodological heterogeneity, SWD was selected as the effect amount. Because of the significant heterogeneity between the two studies (*P* < 0.00001, *I*^2^ = 96%), a random-effect model was used.  Figure 18 shows that the difference is not statistically significant (SMD = −1.38, 95% CI [−3.37, 0.61], *P*=0.17). It has not been proven that there is a statistically significant difference in improving CRP between oral SP alone and sulfasalazine.

#### 3.5.3. Total Effective Rate

Both studies [49, 50] were included and reported a total effective rate. No pooled analysis was performed due to inconsistent efficacy criteria. The results of the two studies showed that there was a significant difference (*P* < 0.05), suggesting that oral SP alone might be more beneficial in the total effective rate of AS patients than sulfasalazine.

### 3.6. Adverse Reactions

Ten studies [39–45, 47, 49, 50] reported adverse reactions. Two other studies [46, 48] did not report any information on adverse reactions. Adverse reactions included skin allergic reactions (rash or itching), digestive symptoms (gastrointestinal upset), systemic symptoms (headache or dizziness), abnormal laboratory indicators (liver and kidney dysfunction, elevated transaminases, decreased white blood cell count, or red blood cell urine), and drug allergy. Due to the wide variety of adverse reactions and the lack of consistent criteria for adverse reactions, no meta-analysis was performed. Therefore, we only conducted a descriptive analysis. In 7 studies [39–45] using oral SP + CPT versus CPT, 17 skin allergic reactions, 15 digestive symptoms, 0 systemic symptoms, 13 abnormal laboratory indicators, and 2 drug allergies (allergy to SP) occurred in the experimental group, and 8 skin allergic reactions, 7 digestive symptoms, 1 headache, 15 abnormal laboratory indicators, and 1 drug allergy (allergy to SSZ) occurred in the control group. In one study [47] using injectable SP + CPT versus CPT, 5 skin allergic reactions and 1 dizziness occurred in the experimental group, and no adverse reactions occurred in the control group. In 2 studies [49, 50] using oral SP alone versus CPT, 4 skin allergic reactions, 3 digestive symptoms, and 1 headache occurred in the experimental group, and 9 skin allergic reactions, 3 digestive symptoms, and 4 abnormal laboratory indicators occurred in the control group. In two studies [41, 44], patients withdrew from the study due to gastrointestinal reactions or allergic reactions. In other studies, adverse reactions were relieved or disappeared after discontinuation or symptomatic treatment. None of the 10 RCTs found statistically significant differences between the experimental group and the control group. See Table 3 for details.

## 4. Discussion

### 4.1. Summary of Evidence for the Efficacy and Safety of Sinomenine

In this study, it has proven that oral SP + CPT may be more effective in treating AS compared with CPT alone in many indicators including BASDAI, morning stiffness time, Schober test, occipital wall test, finger-to-ground distance, 15 m walking time, CRP, and total effective rate, but it has not proven in BASFI, chest expansion, and SER. One study [47] showed that injectable SP + CPT may be more effective compared with CPT alone in BASFI, but no statistical significance in other indicators due to the insufficient number of studies included. It has also proven that oral SP alone may be more effective in improving morning stiffness time compared with CPT. Besides, there are no significant differences in adverse reactions between the experimental group and the control group, which indicates that SP may have good safety in the treatment of AS. Due to the insufficient number and low methodological quality of the included RCTs, it is impossible to carry out more multicenter, large-sample, and high-quality RCTs to further verify the conclusions.

Some scholars believed that the action of sinomenine on the mitogen-activated protein kinases (MAPKs) pathway could inhibit lipopolysaccharide-induced intercellular cell adhesion molecule-1 (ICAM-1) synthesis, which revealed the protective effect of sinomenine on postinflammatory vascular endothelium [27]. Besides, COX is an important rate-limiting enzyme in inflammation. It has two isoforms including COX-1 and COX-2. Generally, drugs exert their anti-inflammatory effects mainly by inhibiting COX-2. However, inhibition of COX-1 can cause adverse reactions. Traditional NSAIDs, such as ibuprofen and indomethacin, cannot selectively inhibit COX-1 and COX-2. The study [51] has found that sinomenine has a weak inhibitory effect on COX-1 enzyme activity within a certain concentration range and does not show a significant dose-effect relationship. Correspondingly, sinomenine has a stronger inhibitory effect on COX-2 enzyme activity. Therefore, sinomenine may selectively inhibit COX enzyme activity. And the chemical structure of sinomenine is similar to that of morphine, which has mild sedative and analgesic effects. Studies have confirmed that sinomenine can treat acute and chronic pain after inflammation and nerve injury, and there are no sedative and other side effects within the effective dose range [28]. This may be one of the reasons why sinomenine is effective in treating AS without obvious adverse reactions.

### 4.2. Limitations and Applicability of the Systematic Review and Meta-Analysis

The high risk of bias in these RCTs was mainly due to inappropriate trial design and selective reporting. Serious inaccuracies are due to the small sample size of the included studies. The included studies were all published in Chinese and unpublished grey literature might not be retrieved, so the existence of publication bias could not be excluded. Therefore, the overall methodological quality of the included studies is low, which reduces the strength of evidence recommendation of this systematic review and meta-analysis. In the future, it is still necessary to carry out more multicenter, large-sample RCTs following the requirements of the SPIRIT 2013 statement (Defining Standard Protocol Items for Clinical Trials) [52] to further verify the conclusions. At the same time, we should pay more attention to the design and evaluation of clinical trials, improve the quality of evidence, use internationally accepted diagnostic criteria, and outcome measures that are closely related to efficacy [53].

Besides, the applicability of meta-analysis results was limited due to the reasons below. On the one hand, the follow-up time of the included RCTs was from one month to 18 months. Considering that the main purpose of our study is to observe whether SP is effective in patients with AS, therefore, the relationship between the follow-up time and the efficacy has not been explored in depth. In the future, more large samples and high-quality RCTs with the same follow-up time will be integrated to provide evidence of the time-effect relationship. On the other hand, in China, the commonly used symptom outcomes are morning stiffness time, the Schober test, chest expansion, occipital wall test, finger-to-ground distance, and 15 m walking time. However, these indicators are easily affected by the subjective consciousness of patients and evaluators. BASDAI and BASFI are internationally recognized symptom scales. As these scales are not widely used and promoted in China, only a few of the included RCTs use them. The unification and standardization of outcomes are important for effective and high-quality information mining based on a large number of clinical trials. Therefore, establishing the core outcome set of various diseases is the first problem that needs to be solved.

### 4.3. Prospects

Traditional Chinese medicine culture has been passed down in China for thousands of years. Chinese herbal medicine is an important part of traditional Chinese medicine culture. Chinese herbal medicine is not only rich in sources and low in price but also has great potential and research significance in the treatment of various diseases. By looking at the different cost areas within outpatient costs, Kirchhoff et al. found that medication costs played an important role, contributing approximately a third of the overall amount [54]. Currently, tumor necrosis factor inhibitor (TNFi) is a strongly recommended drug for the treatment of adults with active AS, even if an NSAID has been used [12]. However, the high cost of TNFi brings a heavy economic burden to families and society. SP is a Chinese herbal medicine extract, and its production cost is much lower than that of chemical drug synthesis. In the future, it is necessary to evaluate the cost-benefit of SP versus TNFi in the treatment of AS to provide more reliable evidence for further research and clinical decision making.

## 5. Conclusions

In summary, this study shows that the combination of oral SP on the basis of CPT is more effective than CPT alone and its safety is good in the treatment of AS, which indicates that oral SP could be recommended as a potent and promising adjuvant therapy for AS. However, this study cannot provide evidence for the effect of injectable SP on AS due to few studies. In addition, due to the low methodological quality of the included RCTs and the limitations of the meta-analysis, it is still necessary to carry out more multicenter, large-sample, and high-quality RCTs to further verify the conclusions.

## Figures and Tables

**Table 1:** Search strategy in PubMed.

Search		Query	Items found
#1		Sinomenine[Title/Abstract]	403
#2		Sinomenium[Title/Abstract]	151
#3		Zhengqing Fengtongning[Title/Abstract]	9
#4		Zhengqingfengtongning[Title/Abstract]	0
#5		#1 OR #2 OR #3 OR #4	449
#6		Spondylitis, Ankylosing[MeSH Terms]	14294
#7		Ankylosing spondylitis[Title/Abstract]	13433
#8		#6 OR #7	18400
#9		Randomized controlled Trials as Topic[MeSH Terms]	130251
#10		Randomized controlled Trial[Publication Type]	492614
#11		Controlled clinical Trial[Publication Type]	581079
#12		Equivalence Trial[Publication Type]	392
#13		Randomized controlled trial[Title/Abstract]	65358
#14		Random Allocation[MeSH Terms]	100812
#15		Double-Blind Method[MeSH Terms]	153964
#16		Single-Blind Method[MeSH Terms]	27468
#17		Clinical Trial[Publication Type]	839529
#18		Research Design[MeSH Terms]	428214
#19		Placebos[MeSH Terms]	34533
#20		placebo$[Title/Abstract]	207201
#21		random*∗*[Title/Abstract]	1081385
#22		trial$[Title]	206169
#23		#9 OR #10 OR #11 OR #12 OR #13 OR #14 OR #15 OR #16 OR #17 OR #18 OR #19 OR #20 OR #21 OR #22	1854129
#24		#5 AND #8 AND #23	1

**Table 2:** Characteristics of the studies included.

Study ID	Sample size (E/C)	Sex (M/F)		Age (mean ± SD or range)		Duration of disease (Mean ± SD or range)		Intervention		Follow-up time	Outcomes
		E	C	E	C	E	C	E	C		
Liu [39]	36/32	29/7	24/8	24.0 ± 4.8	22.0 ± 5.4	(4.8 ± 3.6) y	(4.5 ± 3.2) y	SP (ZFCT[po, 40 mg, tid]) + CPT	CPT (SSZ)	18 m	①②③④⑤⑥⑦⑧
Liu [40]	30/20	26/4	18/2	15∼39	17∼58	(0.5∼5) y	(0.5∼6) y	SP (ZFCT[po, 80 mg, tid]) + CPT	CPT (SSZ)	3 m	④⑦⑧⑨
Dong [41]	45/34	42/3	32/2	24∼72	26∼72	NA	NA	SP (ZFCT[po, 40 mg, tid]) + CPT	CPT (SSZ + Meloxicam)	24 w (6 m)	①②④⑤⑥⑧⑨
Yang [42]	38/38	31/7	33/5	30.26 ± 13.4	28.67 ± 12.8	(5.31 ± 2.8) y	(6.04 ± 3.1) y	SP (ZFSRT[po, 60 mg, bid]) + CPT	CPT (SSZ)	12 w (3 m)	①②③④⑤⑥⑦⑧⑨⑩
Huang [43]	13/12	NA	NA	NA	NA	NA	NA	SP (ZFSRT[po, 60 mg, bid]) + CPT	CPT (SSZ)	6 m	①②⑤⑦⑧⑨
Yin [44]	31/30	28/6	25/9	26.4 ± 3.5	29.8 ± 2.2	(5.9 ± 1.8) y	(6.1 ± 4.2) y	SP (ZFSRT[po, 60 mg, bid]) + CPT	CPT (SSZ)	12 m	①⑥⑧⑪⑫
Lin [45]	57/58	NA	NA	23.5 ± 13.4	24.7 ± 11.3	3 m∼11 y	5 m∼12 y	SP (ZFSRT[po, 120 mg, bid]) + CPT	CPT (SSZ + NSAIDs + methotrexate)	12 m	①②③④⑤⑥⑦⑧⑨⑩
Yu [46]	40/40	32/8	30/10	27.2 ± 3.0	26.7 ± 3.3	(6.1 ± 1.2) y	(5.7 ± 1.5) y	SP (ZFSRT[po, 120 mg, bid]) +CPT	CPT (SSZ + Thalidomide + Meloxicam)	3 m	①③④⑤⑥⑦⑨⑪
Jie [47]	32/30	NR	NR	NR	.NR	NR	NR	SP (ZFI[intra-articular injection, 35 ml, once a week]) + CPT	CPT (SSZ/Meloxicam)	1 m	②③⑤⑥⑦⑧⑪⑫
An [48]	25/24	19/6	20/4	30.2 ± 8.0	31.3 ± 7.0	(6.3 ± 1) y	(6.9 ± 0.8) y	SP (ZFI[intra-articular injection, 2 ml, qod]) + CPT	CPT (IPT + vitamins + Ranitidine)	1 m	⑦
Liu [49]	60/60	NA	NA	26.5 ± 4.8	26 ± 4.5	6 m∼14 y	6 m∼13 y	SP (ZFSRT[po, 60 mg, bid])	CPT (SSZ)	3 m	①②③④⑤⑥⑦⑧⑨
Chai [50]	28/22	22/6	29/7	28.63 ± 5.23	27.98 ± 3.85	(4.87 ± 1.09) y	(4.91 ± 1.27) y	SP (ZFSRT[po, 120 mg, bid])	CPT (SSZ)	2 m	①②③④⑤⑥⑦⑧

*Note.* E: experimental group; C: control group; M: male; F: female; ZFCT: Zhengqing Fengtongning Conventional Tablet; ZFSRT: Zhengqing Fengtongning Sustained Release Tablet; ZFI: Zhengqing Fengtongning Injection; SSZ: sulfasalazine; NSAIDs: nonsteroidal anti-inflammatory drugs; IPT: inflammatory pain tablet; po: oral preparation; tid: three times a day; bid: twice a day; qod: every other day; y: year; m: month; w: week; NA: not available; ① morning stiffness time; ② Schober test; ③ chest expansion; ④ occipital wall test; ⑤ erythrocyte sedimentation rate (ESR); ⑥ C-reactive protein (CRP); ⑦ total effective rate; ⑧ adverse reactions; ⑨ finger-to-ground distance; ⑩ 15 m walking time; ⑪ Bath ankylosing spondylitis disease activity index (BASDAI); ⑫ Bath ankylosing spondylitis functional index (BASFI).

**Table 3:** Summary of adverse reactions.

Study ID	Experimental group			Control group		
	Sample size	Intervention	Specific case	Sample size	Intervention	Specific case
Liu [39]	36	SP (ZFCT[po, 40 mg, tid]) + CPT	Rash or pruritus: 2; gastrointestinal upset: 3; decreased WBC: 1	32	SSZ	Rash or pruritus: 1; gastrointestinal upset: 3; headache: 1
Liu [40]	30	SP (ZFCT[po, 80 mg, tid]) + CPT	0	20	SSZ	0
Dong [41]	45	SP (ZFCT[po, 40 mg, tid]) + CPT	Gastrointestinal upset: 2; drug allergy (SP): 2	34	SSZ + Meloxicam	Gastrointestinal upset: 2; drug allergy (SSZ): 1
Yang [42]	38	SP (ZFSRT[po, 60 mg, bid]) + CPT	Rash or pruritus: 4	38	SSZ	0
Huang [43]	13	SP (ZFSRT[po, 60 mg, bid]) + CPT	Rash or pruritus: 1	12	SSZ	Liver and kidney dysfunction: 2
Yin [44]	31	SP (ZFSRT[po, 60 mg, bid]) + CPT	Rash or pruritus: 5; gastrointestinal upset: 3; decreased WBC: 2; liver and kidney dysfunction: 1; RBC urine: 4	30	SSZ	Rash or pruritus: 1; gastrointestinal upset: 2; decreased WBC: 2; liver and kidney dysfunction: 2; RBC urine: 3
Lin [45]	57	SP (ZFSRT[po, 120 mg, bid]) + CPT	Rash or pruritus: 5; gastrointestinal upset: 7; elevated transaminase: 5	58	SSZ + NSAIDs + methotrexate	Rash or pruritus: 6; elevated transaminase: 6
Jie [47]	32	SP (ZFI[intra-articular injection, 35 ml, once a week]) + CPT	Rash or pruritus: 5; dizziness: 1	30	SSZ/Meloxicam	0
Liu [49]	60	SP (ZFSRT[po, 60 mg, bid])	Rash or pruritus: 3	60	SSZ	Rash or pruritus: 7; elevated transaminase: 3
Chai [50]	28	SP (ZFSRT[po, 120 mg, bid])	Rash or pruritus: 1; gastrointestinal upset: 3; headache: 1	22	SSZ	Rash or pruritus: 2; gastrointestinal upset: 3; decreased WBC: 1

*Note.* ZFCT: Zhengqing Fengtongning Conventional Tablet; ZFSRT: Zhengqing Fengtongning Sustained Release Tablet; ZFI: Zhengqing Fengtongning Injection; SSZ: sulfasalazine; NSAIDs: nonsteroidal anti-inflammatory drugs; IPT: inflammatory pain tablet; po: oral preparation; tid: three times a day; bid: twice a day; WBC: white blood cell; RBC: red blood cell.

## Abbreviations

AS: Ankylosing spondylitis
BASDAI: Bath ankylosing spondylitis disease activity index
BASFI: Bath ankylosing spondylitis functional index
CI: Confidence interval
CPT: Conventional pharmacotherapy
CRP: C-reactive protein
ESR: Erythrocyte sedimentation rate
IPT: Inflammatory pain tablet
NA: Not available
NSAIDs: Nonsteroidal anti-inflammatory drugs
OR: Odds ratio
RBC: Red blood cell
RCT: Randomized controlled trial
RR: Risk ratio
SMD: Standardized mean difference
SP: Sinomenine preparation
SP + CPT: Sinomenine preparation combined with conventional pharmacotherapy
SSZ: Sulfasalazine
TCM: Traditional Chinese medicine
WBC: White blood cell
WMD: Weighted mean difference
ZFCT: Zhengqing Fengtongning conventional tablet
ZFI: Zhengqing Fengtongning injection
ZFSRT: Zhengqing Fengtongning sustained release tablet.

## Supplementary Materials

Supplementary Materials Annex 1 PRISMA 2009 checklist.
